# The first European consensus on principles of management for achondroplasia

**DOI:** 10.1186/s13023-021-01971-6

**Published:** 2021-07-31

**Authors:** Valerie Cormier-Daire, Moeenaldeen AlSayed, Tawfeg Ben-Omran, Sérgio Bernardo de Sousa, Silvio Boero, Svein O. Fredwall, Encarna Guillen-Navarro, Melita Irving, Christian Lampe, Mohamad Maghnie, Geert Mortier, Zagorka Peijin, Klaus Mohnike

**Affiliations:** 1 Centre of Reference for Constitutional Bone Diseases (MOC), Department of Clinical Genetics, Paris Centre University, INSERM UMR 1163, Imagine Institute, Paris Centre University, Paris, France; 2 Department of Medical Genetics, King Faisal Specialist Hospital and Research Center, Riyadh, Kingdom of Saudi Arabia; 3 Faculty of Medicine, Alfaisal University, Riyadh, Kingdom of Saudi Arabia; 4 Division of Genetic and Genomic Medicine, Department of Medical Genetic, Sidra Medicine and Hamad Medical Corporation, Doha, Qatar; 5 Medical Genetics Unit, Hospital Pediátrico, Centro Hospitalar e Universitário de Coimbra, Coimbra, Portugal; 6 Faculty of Medicine, University Clinic of Genetics, Universidade de Coimbra, Coimbra, Portugal; 7 Department for Limb Lengthening and Axial Correction, Istituto Giannina Gaslini, Genoa, Italy; 8 TRS National Resource Centre for Rare Disorders, Sunnaas Rehabilitation Hospital, Nesodden, Norway; 9 Medical Genetics Section. Department of Pediatrics, Virgen de la Arrixaca University Hospital, IMIB, University of Murcia-UMU, CIBERER-ISCIII, Murcia, Spain; 10 Department of Clinical Genetics, Guy’s and St Thomas’ NHS Foundation Trust, London, UK; 11 Clinic of Neuropediatrics, Epileptology and Social Pediatrics, University Hospital Giessen and Marburg, Giessen, Germany; 12 Department of Pediatrics, School of Medicine IRCCS Istituto Giannina Gaslini, University of Genoa, Genoa, Italy; 13 Department of Medical Genetics, Antwerp University Hospital and University of Antwerp, Antwerp, Belgium; 14 Orthopaedic Surgery and Paediatric Trauma, Hôpital Necker-Enfants Malades, Paris, France; 15 Central German Competence Network for Rare Diseases (MKSE), Dept of Pediatrics, University Hospital, Otto-von-Guericke University, Magdeburg, Germany

**Keywords:** Achondroplasia, Guiding principles, European Achondroplasia Forum

## Abstract

Achondroplasia is the most common type of skeletal dysplasia, caused by a recurrent pathogenic variant in the fibroblast growth factor receptor 3 (*FGFR3)*. The management of achondroplasia is multifaceted, requiring the involvement of multiple specialties across the life course. There are significant unmet needs associated with achondroplasia and substantial differences in different countries with regard to delivery of care. To address these challenges the European Achondroplasia Forum (EAF), a network of senior clinicians and orthopaedic surgeons from Europe and the Middle East representative of the achondroplasia clinical community, came together with the overall aim of improving patient outcomes. The EAF developed a consensus on guiding principles of management of achondroplasia to provide a basis for developing optimal care in Europe. All members of the EAF were invited to submit suggestions for guiding principles of management, which were consolidated and then discussed during a meeting in December 2020. The group voted anonymously on the inclusion of each principle, with the requirement of a 75% majority at the first vote to pass the principle. A vote on the level of agreement was then held. A total of six guiding principles were developed, which cover management over the lifetime of a person with achondroplasia. The principles centre on the lifelong management of achondroplasia by an experienced multidisciplinary team to anticipate and manage complications, support independence, and improve quality of life. There is focus on timely referral to a physician experienced in the management of achondroplasia on suspicion of the condition, shared decision making, the goals of management, access to adaptive measures to enable those with achondroplasia to access their environment, and the importance of ongoing monitoring throughout adolescence and adulthood. All principles achieved the 75% majority required for acceptance at the first vote (range 91–100%) and a high level of agreement (range 8.5–9.6). The guiding principles of management for achondroplasia provide all healthcare professionals, patient advocacy groups and policy makers involved in the management of achondroplasia with overarching considerations when developing health systems to support the management of achondroplasia.

## Background

Achondroplasia is the most common form of skeletal dysplasia, with an estimated prevalence of 3.72–4.6 per 100,000 births [1, 2]. It is caused by a recurrent pathogenic variant in the fibroblast growth factor receptor 3 (*FGFR3*) and is characterised by disproportionate short stature, macrocephaly, frontal bossing, mid-face hypoplasia and trident-shaped hands [3, 4]. Complications of achondroplasia may include spinal stenosis, thoracolumbar kyphosis, genu varum, sleep apnoea, obesity, and otitis media, among others, with compression of the cranial junction often requiring intervention in early infancy [5–7].

The multifaceted nature of achondroplasia requires that many specialties are involved in the ongoing care, an arrangement that varies between countries. Differences exist in the lead physician responsible for overall patient management (paediatric endocrinologists, clinical geneticists, surgeons), methods for prenatal diagnosis, use of limb lengthening, use of MRI scanning in infants, structure of the multidisciplinary team (MDT), systems of referral, differing roles of patient support groups, and guidelines for management (or lack thereof) at country- and centre-level. With specialists working within their own field of expertise, there has historically been limited interaction between specialists in Europe. With the advent of medical therapy development in achondroplasia, specialists in the condition began coming together through clinical trials and allied Advisory Boards. While individual specialist groups and scientific societies exist, and European Reference Networks, such as the European Reference Network on Rare Bone Diseases (ERN BOND), bring together experts in rare diseases, there is no forum specifically dedicated to achondroplasia and representative of the clinical community who manage it.

### Treatment recommendations

Previously, there have been no universally agreed European recommendations for achondroplasia. There have been published recommendations which include achondroplasia in part, such as prenatal evaluation and delivery of patients with skeletal dysplasia [8], and there is guidance outlined in other areas of the world [9]. Several of the authors are currently involved in the development of detailed international recommendations for the management of achondroplasia.

### The European Achondroplasia Forum

The European Achondroplasia Forum (EAF) came together to address unmet needs in the management of achondroplasia across Europe, with the overall aim of improving patient outcomes. The EAF is a coordinated, independent network of senior clinicians and orthopaedic surgeons from Europe and the Middle East (EUMEA) representative of the achondroplasia clinical community. All members of the EAF had contributed to publications on achondroplasia in recent years and were representative of the countries in the region and the specialties involved in achondroplasia management. The goals of the EAF are to establish and promote collaboration between leading experts, reflect the variety of specialties involved in care, enable cross-country sharing of best practices, facilitate the uptake of recommendations, and support the development of educational programmes.

Based on learnings from other fields, the EAF felt there was a need to develop a consensus on guiding principles of management of achondroplasia. Several members of the EAF are involved in the development of detailed recommendations, however, all members agreed that there is a need for overarching general principles of management, regardless of where the person with achondroplasia is located.

### Scope and purpose of guiding principles for achondroplasia

The guiding principles are to support healthcare professionals, patient advocacy groups, and policy makers providing services for people with achondroplasia. With every country managing achondroplasia somewhat differently, and the availability of resources varying between countries, the guiding principles provide overarching considerations for the management of achondroplasia that can be applied within all individual systems of management. The primary aim when developing the guiding principles was to provide a framework for optimal care for persons with achondroplasia.

## Developing principles of management in achondroplasia

The EAF gathered a group of 13 senior physicians and surgeons, representative of the different specialties involved in achondroplasia management. The group included two paediatric endocrinologists (KM, MM), seven clinical geneticists (VC-D, MI, EG-N, MDS, TB-O, GM, SBdS), two surgeons (SB, ZP), a neuropaediatrician (CL) and a GP specialised in achondroplasia (SF). There was representation from across the EUMEA region including clinicians from Belgium, France, Germany, Italy, Kingdom of Saudi Arabia, Norway, Portugal, Qatar, Spain, and the UK. The members were all experienced in the management of achondroplasia and the complications thereof, and several had experience in developing recommendations. The European League Against Rheumatism (EULAR) standardised operating procedures for the elaboration, evaluation, dissemination and implementation of recommendations [10] were consulted to ensure the process of developing guiding principles was robust.

All members of the EAF group were invited to submit suggestions for guiding principles which were collated and grouped. It was agreed that a target of up to six principles was optimal as too many may result in confusion and lack of implementation [10]. Six draft principles were developed and for each, the literature was reviewed; where no published evidence was available the collective expert opinion of the members of the EAF was considered. The members of the EAF met in December 2020 to discuss the proposed guiding principles. Each principle was presented and scrutinised in detail for content, support from the literature or collective expert opinion, and wording. Anonymous live voting ensured all opinions were accounted for. The group agreed to use the EULAR protocols for acceptance [10]. For a principle to be accepted, a majority of 75% was needed on the first vote. Should this not be reached, the text was revised, with a subsequent ballot requiring a majority of 66% to accept the principle. If this vote was not successful, further amendments were made until a majority of 50% was reached, or the principle was rejected. Finally, each principle was subjected to an anonymous vote on the level of agreement, with options ranging from 1, no agreement at all, to 10, strong agreement. The draft of the manuscript was circulated to all members of the EAF for comment and approval prior to submission for publication.

### Guiding principles for management of achondroplasia

The members of the EAF agreed on six guiding principles (Table 1) which cover management over the lifetime of a person with achondroplasia. The first principle highlights the need for lifelong management by an experienced MDT, emphasising the importance of close monitoring during the first 2 years (A). The following principles highlight the importance of early referral to an experienced achondroplasia physician following diagnosis either in utero or after birth (B), the importance of joint decision-making between the person with achondroplasia and the clinical team (C), and the primary goals of management (D). The final two principles relate to the need for access to adaptive measures and new treatment options (E), and the importance of ongoing monitoring by an experienced MDT throughout adolescence and adulthood, taking into consideration elements of care particular to these life stages (F). After scrutiny of wording all principles achieved the 75% majority required for acceptance at the first vote (range 91–100%) with high levels of agreement (range 8.5–9.6).

**Table 1:** The 2020 EAF guiding principles of management for achondroplasia

Item	Guiding principle	Vote (%)	Level of agreement (mean; range)
A	Achondroplasia is a lifelong condition requiring lifelong management by an experienced MDT, led by physicians/clinicians experienced in achondroplasia management. Close monitoring during the first 2 years of life is critical	92	8.9 (8–10)
B	When a diagnosis of achondroplasia is made or suspected, either in utero or after birth, the family should be referred as soon as possible to a physician experienced in achondroplasia to discuss the prognosis and management of the condition	100	9.3 (8–10)
C	Decisions around management should be made in the MDT setting jointly with the person with achondroplasia and/or their family	100	9.6 (7–10)
D	The primary goals of management are to enable anticipation, identification and treatment of problems, provide education and support to encourage a healthy lifestyle, positive self-esteem and mental health, autonomy and independence	100	9.2 (8–10)
E	Patients should have access to a variety of adaptive measures, support to ensure proper usage and access to approved treatment options as they become available	91	8.5 (5–10)
F	Regular monitoring in adolescence and adulthood should continue under an MDT with expertise in achondroplasia management. Care should include genetic counselling, transition to adulthood, psychosexual well-being and management of pregnancy	100	9.3 (8–10)

#### Achondroplasia is a lifelong condition requiring lifelong management by an experienced MDT, led by physicians/clinicians experienced in achondroplasia management. Close monitoring during the first 2 years of life is critical

Overall, people with achondroplasia have a normal, or near normal life expectancy [9], although some sources indicate mortality related to heart disease between the ages of 25–35 years is 10 times higher in achondroplasia than in the general population, and that in these patients, life expectancy is reduced by 10 years [11]. The complications of achondroplasia range throughout the life stages, with different considerations for management during infancy, childhood, adolescence and adulthood [5, 9, 12–15].

Evidence from the World Health Organisation (WHO) suggests that people with complex conditions can experience fragmented care [16], and that continual, coordinated care from trusted physicians can be beneficial for clinical outcomes [16–18], as well as leading to greater empowerment and adherence to treatment [16]. The role of the MDT in achondroplasia is a vital one, given the range of complications both physical and psychological that may occur across the life stages [19]. The MDT supporting a person with achondroplasia should be experienced in the condition to enable anticipation of complications and to provide appropriate support [6, 20]. A lead coordinator within the MDT is necessary to ensure consistency of care and referral to other members of the MDT as needed. It was recognised that the specialty of the lead physician varies in different health systems and a specific speciality was not proposed, providing the lead clinician is experienced in the management of the condition.

Close monitoring during the first 2 years of life is vital. Unexpected infant death occurs in 2–5% of infants with achondroplasia [9], and life-threatening events occur in 3.8% of infants within the first year of life [12]. Neurologic and respiratory assessments including sleep studies and assessment for weakness in the limbs can enable timely referral and management by an experienced neurosurgical specialist [9]. Complications such as foramen magnum stenosis must be assessed and treated in early infancy [5, 21, 22], and anticipatory guidance in the early years can mitigate against future complications such as fixed thoracolumbar kyphosis [23, 24], hearing impairment and language disorders [9].

#### When a diagnosis of achondroplasia is made or suspected, either in utero or after birth, the family should be referred as soon as possible to a physician experienced in achondroplasia to discuss the prognosis and management of the condition

Misdiagnosis of achondroplasia is common among those without experience of the condition [25]. While the clinical and radiological features of achondroplasia are recognisable, approximately 20% of affected individuals are not identified at birth [26]. Physicians who are not experienced in the condition lack the knowledge to be able to counsel families effectively [18]. Accurate and timely diagnosis of achondroplasia enables effective counselling from specialists expert in the condition, who can provide clear and comprehensive information enabling a family to make informed decisions. Accurate diagnosis also facilitates referral to an experienced physician to enable anticipatory guidance, expectations of the natural history of the condition, treatment options [8, 9], and the timely management of complications, particularly those that occur in early infancy [5, 6, 8]. Referral to patient organisations and psychologists at the point of diagnosis can also be beneficial [6, 9, 18].

#### Decisions around management should be made in the MDT setting jointly with the person with achondroplasia and/or their family

Shared decision making is understood to be a human right [27], however, there is evidence that in addition it can improve decision quality and create significant health benefits such as increased patient satisfaction, confidence, and clinical outcomes [16, 17, 28]. Members of the EAF agreed that this principle is already widely practised by the clinical community. The wording was closely analysed to ensure emphasis on the wider MDT involvement in the decision-making process. Each decision is multifactorial and must include all relevant members of the MDT. For example, a decision to undergo orthopaedic surgery, such as limb lengthening, will require input from the person with achondroplasia, their family, the lead physician managing overall care, the surgeon, a physiotherapist, and an occupational therapist. The psychosocial aspects should also be addressed. Importance was also placed on the involvement of the person with achondroplasia and their family in the decision-making process. While physicians are expert in the condition, parents are expert in their child’s care; support and consultation with the family is a vital step in shared decision-making [18]. The panel felt consideration should also be given to the age at which a child can provide informed consent for procedures. Their opinions should be considered, particularly when the parents may disagree on the best option for management.

#### The primary goals of management are to enable anticipation, identification and treatment of problems, provide education and support to encourage a healthy lifestyle, positive self-esteem and mental health, autonomy and independence

The purpose of this principle was to encapsulate the key aspects of overall achondroplasia care. The anticipation of problems was considered an important inclusion as regular monitoring and identification of problems enables timely management [5, 6, 29]. Education and anticipatory guidance are essential elements of care to enable physical, practical, and emotional support for the person with achondroplasia and their family whether at home, school or at work [9, 29, 30]. The importance of encouraging a healthy lifestyle was included in this principle because obesity can be a problem in achondroplasia; this can be of particular concern during adolescence as people develop greater autonomy over their own lifestyle choices. Education, MDT monitoring and support for healthy eating and lifestyle can ameliorate the effects of weight gain in later years, and can help to support positive self-esteem [6, 9, 31] and body positivity, which is associated with better mental health status [32]. Positive self-esteem can also be promoted through independent living [6], inclusion in social and working environments, and the development of life and career goals [9, 33]. Access to psychological support should be an option throughout the life of a person with achondroplasia, particularly during times of upheaval, including starting school, during adolescence and at times of family crisis such as divorce of the parents [6].

Quality of life is an important consideration throughout the life of a person with achondroplasia, with both children and adults experiencing a lower quality of life physically, emotionally and socially [14, 34, 35]. Adults with achondroplasia are likely to have an impaired quality of life as they age, due to limitations in physical functioning [14, 19, 36]. Lower mental well-being is also prevalent in adults with achondroplasia, with 56% of respondents with achondroplasia in one study diagnosed with a mental illness such as anxiety or depression [19]. Parents of a child with achondroplasia may also have a reduced quality of life and may report their child to have a lower quality of life [32]. Appropriate support for parents and families of a person with achondroplasia is necessary and may in turn enable them to provide strength and support to the child when facing the challenges of achondroplasia in their daily lives [32]. The impact of achondroplasia on quality of life in children, adults, and their families highlights the need for continued monitoring throughout the life stages by an experienced MDT and for mental health care to be prioritised [7, 19].

The wording of ‘autonomy’ and ‘independence’ was discussed at length, with EAF members agreeing that both were relevant and important to include, with ‘autonomy’ described as relating to decision-making and ‘independence’ as the ability to live and function practically by oneself.

#### Patients should have access to a variety of adaptive measures, support to ensure proper usage and access to approved treatment options as they become available

Improving aspects of daily living for people with achondroplasia can benefit their well-being and quality of life. Children with achondroplasia may find some activities of daily life challenging due to musculoskeletal impairments which can prohibit full physical functioning [37], and some may require assistance with self-care such as toileting, dressing, and bathing for longer than children of average stature. Adults’ physical and mental well-being have been shown to be impacted by their physical limitations [14, 36]. Different adaptive needs will be apparent at all ages [5, 9]. Measures that may help a person with achondroplasia to access their environment may include adaptations to the home, toys, clothing, as well as to school or work environments, and to enable driving [6]. Physiotherapists, occupational therapists and surgeons will play an important role in supporting adaptations for people with achondroplasia. ‘Treatment options’ refers not only to medications, but also to advances in other areas. With new medical therapies on the horizon, timely access will be important to widen the options for management.

#### Regular monitoring in adolescence and adulthood should continue under an MDT with expertise in achondroplasia management. Care should include genetic counselling, transition to adulthood, psychosexual well-being and management of pregnancy

Coordinated care from an experienced MDT is necessary throughout the life of a person with achondroplasia, with complications such as cardiovascular conditions, obesity, sleep apnoea, hearing loss, lumbosacral spinal stenosis, pain, and mental health predominant during adolescence and adulthood [5, 9, 13, 14]. Regular monitoring can enable timely intervention. Similar to the MDT in the paediatric setting, a primary coordinator is needed in adulthood to ensure continuity of care. The specialty of the lead physician may vary, but they must be experienced in the management of achondroplasia. The inclusion of transition to adulthood in this principle is an important one, both in a practical sense of how the person with achondroplasia continues to access coordinated care as an adult, but also to address psychosocial well-being and quality of life in adulthood [19].

Pain is an under-diagnosed and under-treated aspect of achondroplasia [38], with prevalence of pain documented in up to 74.5% of adults, however, few visit a pain specialist for support [14]. Chronic pain, that may begin in childhood [9], has been shown to be associated with poor physical function [36, 38], which may result in diminished daily functioning and may impact quality of life. Gait evaluation and subsequent management in childhood can address activity-induced pain [5]. Pain management will be an important factor in the routine monitoring of persons with achondroplasia throughout adolescence and adulthood, particularly as increasing age and decreased ambulation are associated with chronic pain [36, 38].

Focus on a healthy weight and lifestyle is very important during adolescence and adulthood to mitigate potential future complications [6, 9]. Genetic counselling during adolescence is important to encourage understanding of the genetic nature of the condition and the chances of passing the condition on to any future family [8, 9]. Emphasis was placed on the ‘psychosexual well-being’ of persons with achondroplasia, with the wording carefully chosen to encompass all aspects of relationships, gender, sexual health and sexual orientation. ‘Management of pregnancy’ in this principle refers to pregnancy, potential pregnancy and obstetric delivery for women with achondroplasia; thorough evaluation and careful management during pregnancy and delivery is necessary [8].

## Conclusions

The guiding principles of management for achondroplasia provide all healthcare professionals, patient advocacy groups and policy makers involved in the management of achondroplasia with overarching considerations that can be implemented in any healthcare system. The methodology was developed with specific focus on achondroplasia management and the senior physicians and surgeons involved in the process were all experienced in achondroplasia. Although the breadth of the principles may be applicable within other skeletal dysplasias or congenital genetic conditions, this was not a consideration during the development and implementation of the methods and literature search. The guiding principles were developed in line with the goals of the EAF to establish and promote collaboration and enable cross-country sharing of best practices within the European and Middle East region which its members represent. The principles have been developed as a basis for optimal care of achondroplasia that can be applied in conjunction with more specific recommendations provided at regional, country or centre level. Communication and dissemination of the guiding principles of management within the achondroplasia community is an important step in the aim to improve overall patient care. It is important that there is ongoing collaboration with the wider achondroplasia clinical community to scrutinise in greater detail individual aspects of care including, among others, prenatal diagnosis and referral, transition to adult services, surgical and neurosurgical considerations. Further publications with detailed, consensus-based recommendations on key aspects of care are planned. The collective experience of the wider clinical community will be needed to develop more detailed recommendations. The principles of management for achondroplasia will be reviewed in 3 to 4 years, or when there are developments in the field that may change clinical practice, such as the availability of new treatment options.

## Data Availability

Not applicable.

## Abbreviations

EAF: European Achondroplasia Forum
ENT: Ear, nose and throat
ERN-BOND: European Reference Network on Rare Bone Diseases
EUMEA: Europe and the Middle East
MDT: Multidisciplinary team
WHO: World Health Organisation
